# Is therapy-free remission a realistic goal with cladribine tablets in multiple sclerosis? New insights into the mechanism of action and clinical implications of immune reconstitution with cladribine tablets in MS therapy

**DOI:** 10.1007/s00415-025-13530-1

**Published:** 2025-11-27

**Authors:** C. Kleinschnitz, T. Skripuletz, S. Pfeuffer, M. Pawlitzki, P. Rieckmann, I.-K. Penner, J. Knaup, T. Wagner, M. Hübschen, K. Hellwig, R. Pul

**Affiliations:** 1 University Medicine Essen, Neurology, Essen, Germany; 2 Center for Translational Neuro- and Behavioral Sciences, University Medicine Essen, Neurology, Essen, Germany; 3 Medizinische Hochschule Hannover, Klinik Für Neurologie, Hannover, Germany; 4 Universitätsklinikum, Gießen, Germany; 5 Universitätsklinikum Düsseldorf, Klinik Für Neurologie, Düsseldorf, Germany; 6 Neurologische Facharztpraxis, Magdeburg, Germany; 7 InnKlinikum Altötting, Altötting, Germany; 8 Department of Neurology, Inselspital, Bern University Hospital, University of Bern, Bern, Switzerland; 9 COGITO Center for Applied Neurocognition and Neuropsychological Research, Düsseldorf, Germany; 10 Merck Healthcare Germany GmbH, Weiterstadt, Germany; 11 Katholisches Klinikum Bochum, Bochum, Germany

**Keywords:** Cladribine, Disease-modifying therapy, Immune reconstitution therapy, Memory B cell, Multiple sclerosis

## Abstract

Oral cladribine is a highly effective pulsed selective immune reconstitution therapy (SIRT) that received approval for the treatment of relapsing multiple sclerosis (RMS) in 2017. The concept of SIRT is characterized by brief exposure to active substances with long-term effectiveness, repopulation of lymphocytes, and maintenance of immune competence. In consequence, cladribine tablets allow patients to enter a prolonged treatment-free period, which offers time windows for family planning and vaccinations. Long-term control of disease activity has been linked to the sustained reduction of memory B cells. Based on more than 17 years of follow-up, the favorable safety profile is characterized by manageable front loading side effects and a low cumulative risk. Overall, therapy with cladribine tablets is associated with a low monitoring burden and leads to high treatment satisfaction. Meanwhile, 15 years after primary results from the pivotal trial were published, a vast amount of new data has emerged, including central effects of cladribine tablets. This narrative review discusses existing and emerging efficacy and safety data for cladribine tablets in MS and links these learnings to different patient profiles encountered in clinical practice. These include young patients with newly diagnosed RMS, young patients with highly active disease, and older patients switching from anti-CD20 antibodies or spingosine-1-phosphate modulators.

## Introduction

Oral cladribine is a highly effective pulsed selective immune reconstitution therapy that received approval for the treatment of relapsing multiple sclerosis (RMS) in 2017. Since then, long-term clinical experience has been gained in a large number of people with MS, and real-world data confirmed the positive benefit-risk profile established in clinical trials. By June 2025, a total of 131,017 patients, amounting to 367,021 patient-years, have been treated with cladribine tablets since market authorization [Merck, Data on file]. Unlike maintenance treatments, immune reconstitution therapies (IRTs) use short dosing periods to induce long-term immunological changes. As such, a full treatment course of cladribine tablets comprises two treatment cycles of eight to ten treatment days given one year apart, followed by two treatment-free years. At its core, immune reconstitution describes the restoration of a fully competent immune system following one or more cycles of lymphocyte depletion. During the subsequent reconstitution phase, the immune system recovers; immune competence—defined as the ability to fight infections (including opportunistic ones), generate a strong vaccine response, and perform tumor surveillance—is regained. Cladribine is considered a selective IRT (SIRT) because its depleting effect is limited mainly to B and T cells, with a significantly stronger impact on B cells. The therapeutic effect is hypothesized to be driven by the combined benefit of reducing pathogenic lymphocytes and the emergence of a naive lymphocyte population during regeneration. These long-term qualitative changes in immune function are considered the prerequisite for sustained efficacy [1].

Originally developed as continuous treatment, approval was based on the results of the randomized controlled trial CLARITY (NCT00213135) in patients with active relapsing–remitting MS [2, 3]. Compared to placebo, the later approved dose of cladribine tablets (3.5 mg/kg) demonstrated benefits in terms of significant reductions in annualized relapse rate (ARR) at 96 weeks, risk of 3-month sustained disability progression, and lesion counts on brain magnetic resonance imaging (MRI). The most commonly reported adverse event (AE) was mild (800–1000 × 10^9^ cells/L) to moderate lymphopenia (500–800 × 10^9^ cells/L), an anticipated consequence due to the pharmacological properties of cladribine and the selective depletion of lymphocytes [3]. The results of the CLARITY Extension trial (NCT0064153) provided evidence of long-term disease control without further treatment cycles in year 3 and 4 and a consistent safety profile [4], leading to approval and an increased use in clinical practice. The SIRT concept was further verified by real-world data showing an extended period free of disease activity after the initial treatment courses [5–9]. Meanwhile, 15 years after primary results from CLARITY were published [3], a vast amount of new data has emerged. This narrative review discusses new data of cladribine tablets and how to apply them to patient care in practical cases.

## Long-term treatment-free disease control due to unique mode of action

Migration of lymphocytes into the CNS is a critical event in the pathogenesis of MS, which leads to immune-mediated inflammation and damage of myelin and neurons [10]. Cladribine’s mode of action involves selective depletion of dividing and non-dividing T and B cells, thus limiting the number of lymphocytes transitioning into the CNS. Deoxycytidine kinase (DCK) is required for the phosphorylation of cladribine to its active metabolite 2-chlorodeoxyadenosine triphosphate (Cd-ATP), while 5’-nucleotidase degrades Cd-ATP. In dividing cells, Cd-ATP impairs DNA synthesis. In resting cells, cladribine causes DNA single-strand breaks, rapid nicotinamide adenine dinucleotide consumption, ATP depletion and cell death [11]. Lymphocytes are particularly susceptible to apoptosis due to their relatively high DCK/5′-nucleotidase ratio, favoring the accumulation of Cd-ATP. In consequence of variations in the expression levels of DCK and 5’-nucleotidase between immune cell subtypes, cells of the innate immune system are less affected by cladribine treatment than those of the adaptive immune system [12].

**Fig. 1:**
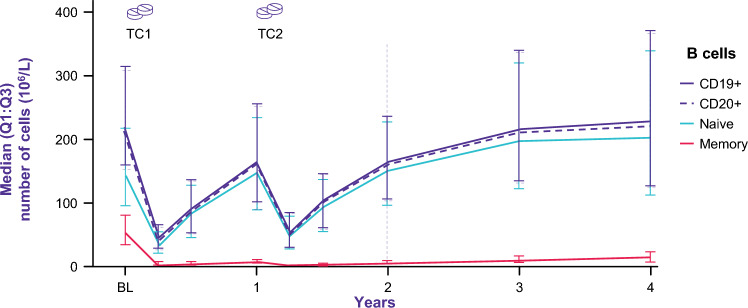
Long-term effect of cladribine tablets on memory B cells. BL, baseline; TC, treatment course. Each treatment course consists of 2 treatment weeks, one at the beginning of the first month and one at the beginning of the second month of the respective treatment year. Each treatment week consists of 4 or 5 days on which a patient receives 10 mg or 20 mg (one or two tablets) as a single daily dose, depending on body weight

Intake of cladribine tablets triggers a rapid and prominent reduction of CD20 + and CD19 + B cells. Additionally, CD3 + T cells with the respective CD3 + CD4 + and CD3 + CD8 + T cell subsets as well as NK cells are significantly reduced 2 weeks after treatment initiation [13]. The less severe reduction of regulatory T cells compared to other T cell subtypes is important to prevent excessive immune reactions of T cells, which could trigger the development of secondary autoimmune diseases [12]. Analyses of pooled data from the CLARITY trial, CLARITY Extension trial and the PREMIERE registry (long-term CLARITY cohort; NCT01013350) to characterize long-term changes in lymphocyte counts in peripheral blood, indicated that recovery of absolute lymphocyte counts (ALC) to the normal range begins soon after treatment in each of years 1 and 2. Interestingly, repopulation dynamics differed for various lymphocyte subsets. After a moderate and temporary reduction in T cell numbers, median CD4 + T cell counts recovered to threshold values (0.35 × 10^9^ cells/L) approximately 43 weeks after the last dose of cladribine tablets in year 2, while median CD8 + cell counts never dropped below the threshold value of 0.20 × 10^9^ cells/L [14]. Regulatory T cells increased by 24 weeks after each cladribine tablets course, which may contribute to long-term immune control [15]. In addition, a rapid decrease in B cell numbers was followed by repopulation reaching threshold values (0.10 × 10^9^ cells/L) within 30 weeks after the last cladribine tablets dose in year 2 [14]. Immunophenotyping revealed the reduction of memory B cells to be most profound and persistent up to 7 years [16–19]. At 24 months, memory B cells remained reduced by 89% from baseline. This effect was maintained over 48 months (Fig. 1) [15, 20]. It is not yet clear how long this reduction will last. The observed change in the B cell subset composition correlated with clinical and cranial MRI disease activity [21]. Long-term data confirmed control of disease activity even beyond year 4 [22, 23]. The actual proportion of patients maintaining freedom of disease activity without additional treatment varies between studies, due to different follow-up periods and endpoints (relapse activity, progression independent of relapses, confirmed disability accumulation, no evidence of disease activity) used for assessment (range 48% relapse-free after a median time of 10.9 years since the last dose to 90% relapse-free in year 5) [22–24]. Further research and a consensus definition of remission are required in this context. Control of disease activity has been linked to the sustained reduction of memory B cells [25]. This longevity of the memory B cell depletion far beyond the administration period is therefore considered a key mechanism of SIRT [16]. These observations were supported by data on the transcriptome and proteome level, indicating a reduction of possibly disease-relevant clones in the memory B cell subset without disrupting the overall clonal composition of B cells [21, 26]. The concept of SIRT was further supported by sustained reductions of the fluid biomarker serum neurofilament light chain (sNfL), which is indicative of intrathecal neuroinflammation and neuroaxonal damage [27].

## Increasing evidence for effects in the CNS

**Fig. 2:**
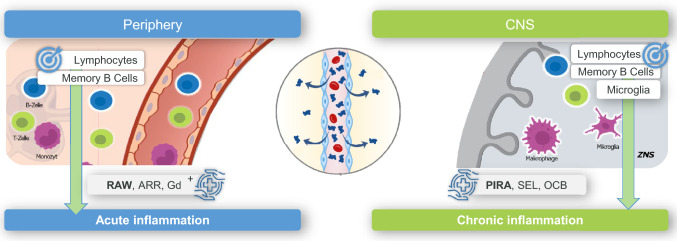
Cladribine tablets trigger effects in both the peripheral and the central nervous system. ARR, annualized relapse rate; Gd +, gadolinium-enhancing lesion; OCB, oligoclonal band; PIRA, progression independent of relapse activity; RAW, relapse-associated worsening; SEL, slowly expanding lesion

Underlying pathologies for progressive MS are located in the CNS. These include focal MS lesions, B cell rich lymphoid aggregates in the meninges, widespread diffuse microglial inflammation and astrogliosis throughout the CNS white matter, as well as age-related neurodegeneration [28]. Targeting these sites of inflammation, demyelination and neuroaxonal damage require penetration of the blood–brain barrier (BBB) [29]. Presence of 25% of serum cladribine in the cerebrospinal fluid (CSF) indicated that cladribine is actually able to pass the BBB into the CNS [30–32], where it affects pathological parameters of central inflammation (Fig. 2) [33, 34]. The observed neutralization of lymphocytes in the CSF corresponds to a decrease in inflammatory markers as evidenced by reductions of oligoclonal bands (OCBs) [27, 35], kappa-free light chains (K-FLC) [33], as well as memory B cells [33, 36, 37]. In fact, the first study to demonstrate that cladribine tablets have sustained effects on reducing intrathecal antibody production was a prospective, longitudinal study with cladribine tablets over 96 weeks, showing a sustained reduction of K-FLC in serum and the CNS. This mirrors a decrease in total intrathecal IgG and B cell chemoattractant CXCL-13 production in the CSF. As the study was limited by the small sample size (*N* = 10) and study duration of 96 weeks, further studies are warranted to confirm the effect on intrathecal antibody production and its correlation to effectiveness [33]. Concurrently, the MAGNIFY-MS study confirmed reductions of K-FLC and IgG indices from baseline at Month 24 [27]. Preclinical studies also indicated an effect on microglia [38, 39]. Taken together, these results suggest potential effects of cladribine within the CNS itself.

The reduction and disappearance of OCBs in cladribine tablets-treated patients was observed in parallel with improvements in the clinical parameters no evidence of disease activity (NEDA)−3, Timed 25-Foot Walk Test (T25FW), Nine-Hole-Peg-Test (9HPT) [27], and Symbol Digit Modalities Test (SDMT) [40]. Whether these clinical benefits are a result of reduced intrathecal inflammation or achieved through peripheral effects needs to be further elucidated. Improved parameters of cognition were observed in the studies MAGNIFY-MS (NCT03364036), CLARIFY-MS (NCT03369665) and CLARIFY-MS Extension (NCT04776213). In MAGNIFY-MS, 43% of patients achieved a clinically relevant improvement in cognitive processing speed by 4 SDMT score points and 45% of patients showed stable cognitive processing speed at Month 24 [41]. This improvement was durable as demonstrated in year 4 of the MAGNIFY-MS Extension. A proportion of 51.6% of patients achieved a 4-score improvement, which is clinically relevant on the group level, and 29.7% achieved an 8-score improvement, reflecting clinical relevance on the individual level [42]. Results from the CLARIFY-MS parent study demonstrated significant improvement in the cognitive function domain of the MSQoL-54 at 24 months [43]. Additionally, preserved mental processing speed as well as verbal and visuospatial memory in years 3 and 4 was observed in the CLARIFY-MS Extension study [44].

Furthermore, depletion of CSF-resident lymphocytes corresponds to a decrease in brain damage as evidenced by less brain atrophy: two years after cladribine tablets initiation, significant treatment effects were observed in the thalamus, corpus callosum, as well as grey and white matter [45, 46]. A subgroup analysis of MAGNIFY-MS by SDMT score at baseline (< 53.5 vs. > 53.5) revealed a positive correlation between preserved brain volume of gray and deep gray matter and SDMT scores [47]. Consistently, a decrease in the incidence rate ratio of paramagnetic rim lesions (PRLs) has been observed after complete treatment with cladribine tablets [48]. PRLs are a subset of MS lesions that remain active in the post-active phase and which are associated with significant degeneration of deep gray matter structures [49, 50]. This outcome is also reflected in low rates of progression independent of relapses (PIRA) observed at month 24 (> 93%) [27, 51]. Furthermore, and in line with post hoc data from CLARITY Extension [52], real-world data confirm sustained effects on EDSS in years 3 and 4 following cladribine tablets initiation. Most patients were stable (54% in year 3, 57% in year 4), some got worse (16% in year 3, 14% in year 4), and about 30% improved [53]. In year 4 after the last dose of cladribine tablets, a total of 79.2% of patients from the MAGNIFY-MS Extension reached NEDA-3 (absence of relapses, confirmed 6-month disability progression, and new T1 Gd + and/or active T2 MRI lesions) [42].

## Impact of the SIRT concept on safety

Overall, cladribine tablets are well tolerated and display a favorable benefit/risk ratio. In line with the SIRT concept and in differentiation from continuous therapies, most AEs occur within the first 6 weeks after initiation of cladribine tablets [9]. Cumulative to July 2024, the safety profile of cladribine tablets remained consistent with findings from the clinical development program [2, 54] and previous safety updates [55]. Table 1 provides a summary of reported adverse events of special interest. As a consequence of CD8 + cell dynamics, which decrease more slowly than B lymphocytes, and often remain within normal range, no confirmed cases of PML related to oral cladribine have been observed [54]. Following selective, transient lymphocyte reduction after administration of cladribine tablets, lymphopenia is an expected adverse event. The risk of severe lymphopenia (< 500 × 10^9^ cells/L) is low (0.10 per 100 patient-years; 95% CI 0.09–0.12) and varies among patients, depending on individual factors such as prior treatment with DMTs, age, comorbidities, and baseline immune status. For instance, pre-treatment with dimethyl fumarate is associated with a profound risk of developing severe lymphopenia and subsequent herpes virus infections [8].The risk of lymphopenia and its subsequent potential for infections, most often herpes zoster, can be mitigated by pre-emptive measures that include treatment initiation at adequately recovered lymphocyte counts, implementation of personalized monitoring strategies during the acute treatment period, and optimization of vaccination strategies [56].

**Table 1:** Cumulative adverse events of special interest (as of 07 July 2024) [65]

	Adjusted reporting rate^a^ per 100 patient-years, (95% CI)
Hypersensitivity (2858 reports)	1.13 (1.09; 1.18)
Serious infections (1270 reports)	0.50 (0.48; 0.53)
Herpes zoster (830 reports)	0.33 (0.31; 0.35)
Liver injury (623 reports)	0.25 (0.23; 0.27)
Malignancies^b^ (397 reports)	0.16 (0.14; 0.17)
Severe lymphopenia^c^ (259 reports)	0.10 (0.09; 0.12)
Seizures (160 reports)	0.06 (0.05; 0.07)
Opportunistic infections (excluding PML^d^ and tuberculosis) (39 reports)	0.02 (0.01; 0.02)
Tuberculosis (35 reports)	0.01 (0.01; 0.02)

AE, adverse event, CI, confidence interval, PML, progressive multifocal leukoencephalopathy

^a^The reporting rate is adjusted for the cumulative duration of patient exposure to cladribine tablets

^b^The spectrum of malignancies resembled the distribution of cancer types seen in the general population, without any clustering of specific tumor types

^c^ < 500 × 10^9^ cells/L

^d^As of 07 July 2024, there were no confirmed cases of PML related to oral cladribine

The SIRT concept allows pregnancy planning during the treatment-free period, whereby females should observe a 6-month safety period between the last dose of cladribine tablets and conception. Due to possible effects on gametogenesis, male patients must take precautions to prevent pregnancy of their partner during cladribine treatment and for at least 6 months after the last dose. The effect of cladribine tablets on exposed pregnancies is being investigated in MAPLE-MS, a 10-year enhanced pharmacovigilance program initiated as a post-marketing requirement for the US Food and Drug Administration (FDA). Based on 157 maternal and 16 paternal exposure pregnancies with known outcomes evaluated in an interim analysis at year 7, the frequency of stillbirths was low, and the majority of pregnancies resulted in live births. Within 7 years, a single major congenital anomaly (atrial septal defect) has been reported to the global patients’ safety database. The outcomes are in line with published estimates from the general population and MS patient cohorts [57].

Due to the recovery of B and T cells, patients treated with cladribine tablets are able to mount an adequate immune response to seasonal influenza vaccination regardless of the duration of cladribine tablets therapy and the time interval since the last dose, as demonstrated in a controlled prospective vaccination study [58]. Similarly, treatment with cladribine tablets did not impair humoral response to COVID-19 vaccination, whereby the time since last cladribine tablets dose, age, prior therapy, lymphocyte count as well as B and T cell counts had no effect on seropositivity of anti-SARS-CoV-2 IgG antibodies [59, 60].

## Cladribine tablets’ position within the MS treatment landscape

Currently, a total of 21 DMTs are approved for the treatment of MS. There are no prospective controlled head-to-head trials comparing cladribine tablets with other high-efficacy therapies. In some highly active cases, disease control may prove challenging to achieve and treatment with anti-CD20 antibodies may be more efficacious. However, in light of continuous immunosuppression with anti-CD20 antibodies and natalizumab, cladribine tablets provide an additional high-efficacy disease-modifying treatment option. The advantages of cladribine tablets include absence of cumulative safety risks inherent to continuous immunosuppression and absence of immunoglobulin deficiency. In particular, cladribine tablets represent a viable therapeutic choice when a high efficacy therapy is required at an early stage in the course of the disease, as well as for subsequent therapy following anti-CD20 treatment at a later stage in the disease course. The concept of SIRT allows patients to enter a prolonged period of treatment-free remission if they achieve complete freedom from disease activity. The length of this status is currently investigated in several real-world cohorts, yet likely depends on individual factors, such as prior disease activity, age, disease duration, and baseline disability. Predictive factors for treatment failure or need for re-treatment are still a subject of investigations. In case of recurring disease activity, additional courses of cladribine can be administered in years 5 and 6. According to the SmPC, lymphocyte counts must be normal (≥ 1000 × 10^9^ cells/L) before initiating treatment in year 1 and at least 800 × 10^9^ cells/L before initiating treatment in year 2 [61]. The same criteria should be applied to redosing with additional courses of cladribine tablets. Real-world data have shown that lymphocytes are usually sufficiently recovered by the time redosing is considered [22]. The treatment-free period and its inherent opportunities for the patient regarding family planning and a life without restrictions due to continuous therapy and possible side effects are a compelling argument in favor of cladribine tablets.

## Implications for clinical practice

The learnings derived from cladribine’s unique mode of action as an immune reconstitution therapy offer a variety of implications for clinical practice when linking them to different patient profiles. Overall, the choice for cladribine tablets over other high-efficacy therapies is not only based on specific clinical or paraclinical parameters, but also on patient-relevant factors, which have gained importance in the treatment decision. These include family planning, the need for a convenient therapy, non-response under anti-CD20 antibodies, and high infection rate under continuous immunosuppression. The following patient profiles depict these scenarios and may serve as guidance in the decision process for cladribine tablets.

### Patient profile 1: Early use in female DMT-naïve patients

A 25-year-old woman with newly diagnosed RMS and factors indicating highly active disease would like to have children before turning 30. She prefers not to expose her child to medication during pregnancy.

MS is most commonly diagnosed in women in primary child-bearing years. Accordingly, treatment choice should consider family planning. According to the SmPCs, treatment with anti-CD20 antibodies should be avoided during pregnancy unless the potential benefit to the mother outweighs the potential risk to the fetus. Some women prefer taking no medication at all during pregnancy; however, discontinuing fingolimod or natalizumab has been associated with a relevant risk of disabling relapses [62, 63]. Therefore, cladribine tablets are a good alternative if a pregnancy is planned. While cladribine tablets are contra-indicated during pregnancy, the treatment-free period after the full dose has been administered in year 2 offers a time window for women to become pregnant without pressure of time under highly effective therapeutic protection during pregnancy and postpartum [64]. In line with the current SmPC, a safety window of 6 months after the last cladribine tablets dose should be observed before conception [61]. In this context, the favorable response to vaccinations during therapy with cladribine tablets is relevant for vaccinations recommended during pregnancy, such as influenza and pertussis [58]. So far, post-approval surveillance data did not indicate any new safety signals in pregnant women. Out of 157 pregnancies with known outcomes, 20.4% resulted in spontaneous abortions and one major (atrial septal defect) and three minor congenital anomalies occurred, which is within the scope observed in the general population (10.0–24.0% and 2.0–4.4%, respectively) [57, 65]. Limited data from case reports have shown that cladribine is excreted in human milk. The quantity is not yet well established; however, plasma concentrations of cladribine decline rapidly, with an effective half-life of approximately 1 day. Because of the potential for serious adverse reactions in breast-fed infants, breast-feeding is contraindicated during treatment with cladribine tablets and for 1 week after the last dose [61]. During the treatment-free period, breast-feeding is possible. If women become pregnant after the first course, breast-feeding should be paused twice when the second course is due to allow administration for 4–5 days and observation of the 1-week safety margin in each of the two administration months, respectively. If women become pregnant in the time window after two courses of cladribine tablets have been completed, the breast-feeding period falls within the treatment-free period and can be executed without risk of exposure. Generally, data indicate beneficial effects of cladribine tablets in terms of delayed disability worsening when initiated at an early stage. In particular, in patients with clinically isolated syndrome (CIS), cladribine tablets effectively reduced the rate of conversion to MS [66, 67].

### Patient profile 2: Early use in DMT-naïve patients

A 29-year-old male has been newly diagnosed with highly active RMS. His job requires frequent traveling, which leaves little time for regular visits to the physician and monitoring appointments. The patient displays adherence issues and needle phobia. He would like to “forget” his disease.

The oral administration route, the convenient dosing regimen and the durable effect after the full dose has been administered make cladribine tablets a good option for patients with adherence problems [68]. Real-world studies have shown that the short administration time totaling 20 days, and oral route of administration of cladribine tablets result in a high degree of adherence and patient satisfaction [9, 69]. The monitoring burden following each treatment phase is low and the treatment-free periods offer time windows for traveling between monitoring appointments. The absence of an active therapy is reflected in improved quality of life scores over 2 years compared to baseline [43].

### Patient profile 3: Switch from anti-CD20 antibodies due to non-response

A 32-year-old woman fails to respond to a therapy with anti-CD20 antibodies.

Anti-CD20 antibodies are usually highly effective in people with MS. Yet, effectiveness and tolerability are limited in a small proportion of patients for various reasons. Recent data indicate that patients showing signs of chronic or acute inflammatory disease activity under anti-CD20 therapies may benefit from a switch to cladribine tablets [70–74]. Out of 30 patients from a retrospective analysis, who switched from anti-CD20 therapies to cladribine tablets, 60% achieved NEDA-3 under the second therapy [74]. Similarly, presence of MRI activity declined from 44% to ≤ 17% and ARR decreased from 0.35 to ≤ 0.18 after switching from another high-efficacy therapy to cladribine tablets [72]. Consistently, retrospective data from the US showed that all patients switching from natalizumab or ocrelizumab to cladribine tablets were relapse-free and free of MRI activity in years 2 (natalizumab, n = 25; ocrelizumab, n = 14) and 3 (natalizumab, n = 10; ocrelizumab, n = 8). The annualized relapse rate (ARR) at baseline (initiation of cladribine tablets) was 0.16 (n = 37) and 0.28 (n = 32) after prior natalizumab and ocrelizumab therapy, respectively. A total of 86.4% and 84.4% had been free of MRI activity at baseline, respectively [70]. The obtained benefit after switching from anti-CD20 antibodies may be ascribed to the capability of cladribine tablets to penetrate the blood–brain barrier and affect pathological parameters of central inflammation [33, 34]. Switching to natalizumab would also be an option. However, in case of John Cunningham virus (JCV) positivity or high JCV antibody index, this is usually avoided in clinical practice.

### Patient profile 4: Switch from anti-CD20 antibodies due to increased infection risk

A 55-year-old woman received anti-CD20 therapies over 7 years with good results in terms of disease control. During the last year she repeatedly suffered from infections and hypogammaglobulinemia.

Meanwhile, patients aged 45–65 years form a large group among MS patients [75]. The risks in elderly patients must be taken into account during any therapy. As a consequence of continuous B cell depletion during prolonged treatment with anti-CD20 antibodies and coupled with immunosenescence, there is an increased risk of serious infections in the elder population [76]. Current studies examine the feasibility of discontinuing therapy after 5 years of maintained stable disease control. Whereas the randomized clinical trials DISCOMS and DOT-MS indicated the potential risk of disease reactivation following treatment discontinuation, both investigated mainly patients on platform therapies [77, 78]. A French observational study examining discontinuation of high efficacy DMTs found that discontinuation was associated with a significantly higher relapse risk (Hazard ratio of 4.1), especially for natalizumab and fingolimod. Patients stopping anti-CD20 antibodies did not experience increased relapse rates within a mean follow-up time of 3.0 years after discontinuation [79]. Consistently, a multicenter retrospective study indicated a rate of disease recurrence < 10% within a short observation period of 1.5 years after discontinuing ocrelizumab [80]. However, resuming treatment is indicated when disease activity returns. Cladribine tablets are the only MS therapy that included people with MS up to the age of 65 years in its pivotal study CLARITY (NCT00213135). A subgroup analysis of CLARITY confirmed that cladribine tablets were highly efficacious (defined as relapse reduction greater than 50% from baseline) in patients > 40 years [81]. Superior relapse control was shown in a propensity score matched analysis from the international longitudinal MS registry MSBase in patients > 50 years starting cladribine tablets compared to DMT continuation [82]. This circumstance together with the favorable efficacy and safety profile makes cladribine tablets a viable option after anti-CD20 therapies or as exit strategy for MS patients of age. Hypogammaglobulinemia is a side effect observed under anti-CD20 therapy, whereas IgG titers as a marker of immune competence have been shown to remain stable up to 4 years in patients receiving cladribine tablets [1, 14, 83–88]. Concordantly, data from an Australian single center cohort demonstrated increased IgG levels after switching older patients (mean age 53 years) after a mean duration of 17 months from ocrelizumab to cladribine tablets [71]. These observations are backed by a recent study investigating the switch from anti-CD20 therapies to cladribine tablets. Prevalent IgG deficiency resolved in most cases within 6 to 12 months after switching to cladribine tablets [74].

Neutropenia is another side effect during CD20-depleting therapies that may trigger a decision to switch therapies [89]. A reduction in neutrophils has also been observed during therapy with cladribine tablets, but not below the normal range [90].

### Patient profile 5: Switch from S1P receptor modulators due to increased infection risk

A 52-year-old woman taking fingolimod over 10 years achieved adequate disease control. The frequency of infections increased over the last 2 years.

Similarly, an increased risk of infections is observed in aging patients receiving S1P receptor modulators. Data from the US showed reduced ARR in years 1–3 following a switch from S1P receptor modulators to cladribine tablets, whereby no lymphopenia issues were observed [91]. The transition from fingolimod to another DMT requires careful management because its discontinuation can trigger rapid lymphocyte redistribution, leading to increased inflammation and rebounds [92]. Key risk factors include younger age, prolonged use of fingolimod, and extended washout periods, with the highest relapse risk typically occurring within the first 3–6 months following discontinuation [93–95]. Zhou et al. have reported a higher frequency of relapses when switching from fingolimod to cladribine tablets compared to ocrelizumab and natalizumab, with a median washout period of 40 days [96]. Nygaard et al. have mentioned that 7 of 33 patients experienced disease activity, which they classified as rebound. The washout time between fingolimod and cladribine tablets was 44 ± 31 days [97]. Because the effect of cladribine tablets starts with a certain delay, the question arises as to the optimum wash-out time. A French expert opinion recommended initiating cladribine tablets within two weeks of fingolimod cessation to mitigate inflammatory rebound [98]. The employment of a 2-week washout period is supported by results from the FinClad study that evaluated the safety and short-term efficacy of cladribine tablets in people with MS who were discontinuing fingolimod due to elevated liver enzyme levels. A longer washout period was significantly associated with the presence of disease activity [99]. The risks of omitting a washout period or even overlapping the therapy for two weeks remain unclear.

## Key benefits of cladribine tablets

The expanded treatment landscape includes many options for highly active MS, allowing a personalized approach according to the needs of each individual patient. The choice of treatment should be based on a shared decision-making process including factors such as disease activity, benefit–risk profile, and patient preference [100]. In addition, potential treatment sequences in case of response failure or tolerability issues should be considered. The key benefits of pulsed immune reconstitution therapy with cladribine tablets comprise brief exposure to active substances with long-term effectiveness, repopulation of lymphocytes, and maintenance of immune competence. Cladribine tablets deliver high flexibility with a potential for extended treatment-free periods and unrestricted long-term management options. The extended treatment-free period offers a window of opportunity for vaccinations and pregnancies. Based on more than 17 years of follow-up, the favorable safety profile is characterized by manageable front loading side effects and a low cumulative risk. Therapy with cladribine tablets is associated with a low monitoring burden and leads to high treatment satisfaction [9]. Regarding treatment costs over 4 years, the brief administration period of a pulsed therapy offers obvious economic benefits compared to continuously administered therapies and anti-CD20 antibodies in general. According to an analysis of prescription data, no further treatment costs were due for 63% of patients who did not require treatment between years 3 and 6 following cladribine tablet initiation [24]. The cost-effectiveness has been confirmed in several country-specific models [101–105].

## Conclusion and outlook

Overall, the data confirmed the predicted benefit of cladribine tablets: very short exposure time, maintained competency of immune system, high efficacy of cladribine tablets even beyond year five, good safety profile with well characterized and manageable side effects linked to the intake phases and low cumulative risk, and low burden of treatment and monitoring. Key characteristics that distinguish cladribine tablets from other high-efficacy DMTs are summarized in Table 2. Of note, some of the data presented here are limited by small patient numbers and non-peer-reviewed publications. Confirmation in peer-reviewed journals is eagerly awaited within the next few years. Options for cladribine tablets in DMT-naïve patients with radiological signs plus paraclinical markers according to the new McDonald criteria 2024 [106], as an exit strategy, and in SPMS are currently being explored. The duration of the treatment-free period varies between individuals, depending on factors such as disease activity before cladribine tablets initiation and prior DMTs. How far the treatment-free period can be stretched remains to be established as more data become available with each additional year following cladribine tablets initiation. However, a subset of patients may not achieve sufficient disease control and show signs of ongoing inflammatory activity, requiring either additional cladribine tablets treatment or a timely switch to an alternative high-efficacy therapy. Additional open research questions include predictive biomarkers for treatment response. In this context, preliminary 24-month data from MAGNIFY-MS showed that cladribine tablets effectively reduced peripheral biomarkers GFAP and NfL. A trend toward association of CNS intrathecal activity markers CXCL-13 and K-FLC indices with clinical outcomes 6-month confirmed disability progression (6mCDP), PIRA, and relapses was observed. However, since very few patients experienced 6mCDP, PIRA, or relapses, the statistical power to detect significant associations was reduced, warranting confirmation in larger populations [107]. Long-term safety data up to 8 years have been captured in the prospective PREMIERE registry, which ran from 2009 to 2018 and included patients who had completed the RCTs CLARITY and CLARITY Extension. Long-term efficacy data are captured in CLASSIC-MS, of which follow-up data of 10.9 years (median) are available so far. As approval was in 2017, no long-term real-world data beyond 10 years are yet available. However, several patient cohorts are continuously monitored to capture data on effectiveness and safety over time. Safety data are continuously reported to the pharmacovigilance department. So far, the positive benefit–risk profile established in clinical trials has been confirmed with the additional patient-years gained in clinical practice.

**Table 2:** Key characteristics that distinguish cladribine tablets from other high-efficacy DMTs

Characteristics	Cladribine Tablets	Other HE-DMTs^a^
Administration schedule	Short exposure time, followed by extended treatment-free period	Continuous therapy
Safety profile	Front-loading, side effects mainly linked to intake phase	Cumulative risk
Immune competency	Maintained	Continuous immunosuppression
Pregnancy	Possible during treatment-free period	Depending on type of DMT possible after benefit-risk considerations (anti-CD20 antibodies, natalizumab) or contraindicated for S1P-RM
Treatment burden	Low	Higher (depending on type of DMT)

DMT, disease-modifying therapy; HE-DMT, high-efficacy disease-modifying therapy; S1P-RM, sphingosine-1-phosphate receptor modulator

^a^Other high-efficacy DMTs include anti-CD20-antibodies, natalizumab, and S1P-RM

## Key clinical take-aways

- Short-course cladribine tablets provide long-term control of disability progression with low rates of PIRA, RAW, and CDA
- Maintained competency of immune system
- Manageable side effects mainly linked to the intake phases and low cumulative risk
- Low burden of treatment and monitoring
- The extended treatment-free period offers time windows for family planning and vaccinations

## Data Availability

No new data were generated or analyzed in the course of this review. All data referenced are available in the cited sources.

## References

[1] Giovannoni G, Mathews J (2022) Cladribine tablets for relapsing-remitting multiple sclerosis: a clinician’s review. Neurol Ther 11:571–595 35318617 10.1007/s40120-022-00339-7 PMC8940595

[2] Cook S, Vermersch P, Comi G, Giovannoni G, Rammohan K, Rieckmann P, Sørensen PS, Hamlett A, Miret M, Weiner J, Viglietta V, Musch B, Greenberg SJ (2011) Safety and tolerability of cladribine tablets in multiple sclerosis: the CLARITY (CLAdRIbine Tablets treating multiple sclerosis orallY) study. Mult Scler 17:578–593 21228029 10.1177/1352458510391344

[3] Giovannoni G, Comi G, Cook S, Rammohan K, Rieckmann P, Soelberg Sørensen P, Vermersch P, Chang P, Hamlett A, Musch B, Greenberg SJ (2010) A placebo-controlled trial of oral cladribine for relapsing multiple sclerosis. N Engl J Med 362:416–426 20089960 10.1056/NEJMoa0902533

[4] Giovannoni G, Soelberg Sorensen P, Cook S, Rammohan K, Rieckmann P, Comi G, Dangond F, Adeniji AK, Vermersch P (2018) Safety and efficacy of cladribine tablets in patients with relapsing-remitting multiple sclerosis: results from the randomized extension trial of the CLARITY study. Mult Scler 24:1594–1604 28870107 10.1177/1352458517727603

[5] Lizak N, Hodgkinson S, Butler E, Lechner-Scott J, Slee M, McCombe PA, Shaw C, Skibina O, Vucic S, Shuey N, Barnett MH, Parratt J, Butzkueven H, Jack D, Fabris J, Kalincik T (2021) Real-world effectiveness of cladribine for Australian patients with multiple sclerosis: an MSBase registry substudy. Mult Scler 27:465–474 32530363 10.1177/1352458520921087 PMC7897790

[6] Butzkueven H, Kappos L, Wiendl H, Trojano M, Spelman T, Chang I, Kasliwal R, Jaitly S, Campbell N, Ho PR, Licata S (2020) Long-term safety and effectiveness of natalizumab treatment in clinical practice: 10 years of real-world data from the Tysabri Observational Program (TOP). J Neurol Neurosurg Psychiatry 91:660–668 32234967 10.1136/jnnp-2019-322326 PMC7279201

[7] Moccia M, Lanzillo R, Petruzzo M, Nozzolillo A, De Angelis M, Carotenuto A, Palladino R, Brescia Morra V (2020) Single-center 8-years clinical follow-up of cladribine-treated patients from phase 2 and 3 trials. Front Neurol 11:489 32625161 10.3389/fneur.2020.00489 PMC7311570

[8] Pfeuffer S, Rolfes L, Hackert J, Kleinschnitz K, Ruck T, Wiendl H, Klotz L, Kleinschnitz C, Meuth SG, Pul R (2022) Effectiveness and safety of cladribine in MS: real-world experience from two tertiary centres. Mult Scler 28:257–268 33975489 10.1177/13524585211012227 PMC8795224

[9] Ziemssen T, Posevitz-Fejfár A, Chudecka A, Cepek L, Reifschneider G, Grothe C, Richter J, Wagner T, Müller B, Penner IK (2024) Evaluation of therapy satisfaction with cladribine tablets in patients with RMS: final results of the non-interventional study CLEVER. Mult Scler Relat Disord 90:105812 39151238 10.1016/j.msard.2024.105812

[10] Piccio L, Rossi B, Scarpini E, Laudanna C, Giagulli C, Issekutz AC, Vestweber D, Butcher EC, Constantin G (2002) Molecular mechanisms involved in lymphocyte recruitment in inflamed brain microvessels: critical roles for P-selectin glycoprotein ligand-1 and heterotrimeric G(i)-linked receptors. J Immunol 168:1940–1949 11823530 10.4049/jimmunol.168.4.1940

[11] Sigal DS, Miller HJ, Schram ED, Saven A (2010) Beyond hairy cell: the activity of cladribine in other hematologic malignancies. Blood 116:2884–2896 20634380 10.1182/blood-2010-02-246140

[12] Baker D, Pryce G, Herrod SS, Schmierer K (2019) Potential mechanisms of action related to the efficacy and safety of cladribine. Mult Scler Relat Disord 30:176–186 30785074 10.1016/j.msard.2019.02.018

[13] Gingele S, Körner GM, Hümmert MW, Seeliger T, Schwenkenbecher P, Jacobs R, Skripuletz T (2002) Rapid onset of effect on various immune cell subpopulations after treatment initiation with cladribine and ocrelizumab. Presented at ECTRIMS. October 26–28, 2022

[14] Comi G, Cook S, Giovannoni G, Rieckmann P, Sørensen PS, Vermersch P, Galazka A, Nolting A, Hicking C, Dangond F (2019) Effect of cladribine tablets on lymphocyte reduction and repopulation dynamics in patients with relapsing multiple sclerosis. Mult Scler Relat Disord 29:168–174 30885375 10.1016/j.msard.2019.01.038

[15] Wiendl H, De Stefano N, Vermersch P, Barkhof F, Montalban X, Achiron A, Hodgkinson S, Chan A, Prat A, Leocan L, Schmierer K, Sellebjerg F, Petit C, Nolting A, Koelbach R, Gardner L, Derfuss T (2025) Durable impact of immune cell reconstitution over 4 years following short course cladribine tablets: Results from MAGNIFY-MS Extension Study. Presented at ACTRIMS 2025 Forum. Feb 27-March 1, 2025

[16] Ceronie B, Jacobs BM, Baker D, Dubuisson N, Mao Z, Ammoscato F, Lock H, Longhurst HJ, Giovannoni G, Schmierer K (2018) Cladribine treatment of multiple sclerosis is associated with depletion of memory B cells. J Neurol 265:1199–1209 29550884 10.1007/s00415-018-8830-y PMC5937883

[17] Wiendl H, Schmierer K, Hodgkinson S, Derfuss T, Chan A, Sellebjerg F, Achiron A, Montalban X, Prat A, De Stefano N, Barkhof F, Leocani L, Vermersch P, Chudecka A, Mwape C, Holmberg KH, Boschert U, Roy S (2023) Specific patterns of immune cell dynamics may explain the early onset and prolonged efficacy of cladribine tablets: a MAGNIFY-MS substudy. Neurol Neuroimmunol Neuroinflamm. 10.1212/NXI.0000000000200048 36411081 10.1212/NXI.0000000000200048 PMC9679889

[18] Holm Hansen R, von Essen MR, Reith Mahler M, Cobanovic S, Sellebjerg F (2024) Sustained effects on immune cell subsets and autoreactivity in multiple sclerosis patients treated with oral cladribine. Front Immunol 15:1327672 38433828 10.3389/fimmu.2024.1327672 PMC10904620

[19] Messner M, Unterhofer M, Strauss J, Mink S, Cadamuro J, Oberkofler H, Hitzl W, Wipfler P, Trinka E, Moser T (2025) Long-term impact of oral cladribine on humoral immunity in multiple sclerosis. Ther Adv Neurol Disord 18:17562864251357276 40766204 10.1177/17562864251357275 PMC12322351

[20] Wiendl H, De Stefano N, Barkhof F, Montalban X, Achiron A, Derfuss T, Chan A, Hodgkinson S, Prat A, Leocani L, Schmierer K, Sellebjerg F, Vermersch P, Jin H, Järvinen E, Chudecka A, Gardner L (2023) Blood biomarker dynamics in highly active relapsing multiple sclerosis patients treated with cladribine tablets: Results of the 2-year MAGNIFY-MS study. Mult Scler 29:21–22

[21] Teschner VE, Fleck AK, Walter C, Schwarze AS, Eschborn M, Wirth T, Steinberg OV, Schulte-Mecklenbeck A, Lu IN, Herrera-Rivero M, Janoschka C, Lünemann JD, Schwab N, Meyer Zu Hörste G, Varghese J, Gross CC, Pul R, Kleinschnitz C, Mader S, Meinl E, Stoll M, Wiendl H, Klotz L (2023) Single-cell profiling reveals preferential reduction of memory B cell subsets in cladribine patients that correlates with treatment response. Ther Adv Neurol Disord 16:17562864231211077 38084102 10.1177/17562864231211077 PMC10710756

[22] Kleinschnitz C, Skuljec J, Kowarik MC, Ernst M, Woitschach L, Cepek L, Rau D, Kühnler B, Schlemilch-Paschen S, Schwab M, Pul R (2025) First insights into the safety and effectiveness of additional courses with cladribine tablets under real-world conditions. Mult Scler Relat Disord 97:106398 40147286 10.1016/j.msard.2025.106398

[23] Giovannoni G, Boyko A, Correale J, Edan G, Freedman MS, Montalban X, Rammohan K, Stefoski D, Yamout B, Leist T, Aydemir A, Borsi L, Verdun di Cantogno E (2023) Long-term follow-up of patients with relapsing multiple sclerosis from the CLARITY/CLARITY Extension cohort of CLASSIC-MS: an ambispective study. Mult Scler 29:719–730 37012898 10.1177/13524585231161494 PMC10176755

[24] Kormann D, Parekh M, von der Maßen K, Huebschen M, Wagner T, Harty G, Alexandri N, Jones M (2025) Treatment continuation with cladribine tablets beyond year 4: analysis of longitudinal prescription data from Germany. Presented at ECTRIMS 2025. September 24-26, 2025

[25] Hodgkinson S, Wiendl H, Barkhof F, X M, Achiron A, Derfuss T, Chan A, Prat A, Leocani L, Schmierer K, Sellebjerg F, Vermersch P, Lehn A, Smyk A, Nolting A, Koelbach R, De Stefano N (2024) Clinical Efficacy of Cladribine Tablets in Patients With Highly Active Relapsing Multiple Sclerosis: 36-Month Interim Results From the Extension Trial to the MAGNIFY-MS Study. Presented at ACTRIMS Forum. 29 Feb - 2 Mar 2024

[26] Ruschil C, Gabernet G, Kemmerer CL, Jarboui MA, Klose F, Poli S, Ziemann U, Nahnsen S, Kowarik MC (2023) Cladribine treatment specifically affects peripheral blood memory B cell clones and clonal expansion in multiple sclerosis patients. Front Immunol 14:1133967 36960053 10.3389/fimmu.2023.1133967 PMC10028280

[27] Schmierer K, Wiendl H, Barkhof F, Montalban X, Achiron A, Derfuss T, Chan A, Hodgkinson S, Prat A, Leocani L, Sellebjerg F, Vermersch P, Jin H, Sponton L, Chudecka A, Gardner L, De Stefano N (2025) Clinical and mechanistic effects of cladribine in relapsing multiple sclerosis: 2-year results from the MAGNIFY-MS study. Ther Adv Neurol Disord 18:17562864251351760 40756532 10.1177/17562864251351760 PMC12317234

[28] Hauser SL, Cree BAC (2020) Treatment of multiple sclerosis: a review. Am J Med 133:1380-1390.e1382 32682869 10.1016/j.amjmed.2020.05.049 PMC7704606

[29] Correale J, Halfon MJ, Jack D, Rubstein A, Villa A (2021) Acting centrally or peripherally: a renewed interest in the central nervous system penetration of disease-modifying drugs in multiple sclerosis. Mult Scler Relat Disord 56:103264 34547609 10.1016/j.msard.2021.103264

[30] Leist TP, Weissert R (2011) Cladribine: mode of action and implications for treatment of multiple sclerosis. Clin Neuropharmacol 34:28–35 21242742 10.1097/WNF.0b013e318204cd90

[31] Liliemark J (1997) The clinical pharmacokinetics of cladribine. Clin Pharmacokinet 32:120–131 9068927 10.2165/00003088-199732020-00003

[32] Kearns CM, Blakley RL, Santana VM, Crom WR (1994) Pharmacokinetics of cladribine (2-chlorodeoxyadenosine) in children with acute leukemia. Cancer Res 54:1235–1239 7906999

[33] Ammoscato F, Skonieczna J, Bestwick J, Holden D, Aboulwafa M, Andrews M, Turner B, Marta M, Schmierer K, Baker D, Giovannoni G, Gnanapavan S (2024) Prospective longitudinal study of Cladribine Tablets (CLADB Study) demonstrates sustained reduction in Kappa Free Light Chains in relapsing multiple sclerosis (RRMS) at 96 weeks (P10-6.008). Neurology 102:3589

[34] Marastoni D, Foschi M, Eccher C, Crescenzo F, Mazziotti V, Tamanti A, Bajrami A, Camera V, Ziccardi S, Guandalini M, Bosello F, Anni D, Virla F, Turano E, Romoli M, Mariotti R, Pizzini FB, Bonetti B, Calabrese M (2024) CSF levels of Chitinase3like1 correlate with early response to cladribine in multiple sclerosis. Front Immunol 15:1343892 38404586 10.3389/fimmu.2024.1343892 PMC10885800

[35] Rejdak K, Stelmasiak Z, Grieb P (2019) Cladribine induces long lasting oligoclonal bands disappearance in relapsing multiple sclerosis patients: 10-year observational study. Mult Scler Relat Disord 27:117–120 30368223 10.1016/j.msard.2018.10.006

[36] Allen-Philbey K, Stephenson S, Doody G, MacDougall A, Aboulwafaali M, Ammoscato F, Andrews M, Gnanapavan S, Giovannoni G, Grigoriadou S, Hickey A, Holden DW, Lock H, Papachatzaki M, Redha I, Baker D, Tooze R, Schmierer K (2025) Effects of cladribine on intrathecal and peripheral B and plasma cells. Clin Exp Immunol. 10.1093/cei/uxae116 39663507 10.1093/cei/uxae116 PMC11748000

[37] Picozza M, Marastoni D, Eccher C, Verdiani A, Anni D, Misiti A, Virla F, Turano E, Camera V, Borsellino G, Battistini L, Calabrese M (2024) Intrathecal B cells in pwMS treated with oral cladribine. Presented at ECTRIMS. September 18–20, 2024

[38] Aybar F, Julia Perez M, Silvina Marcora M, Eugenia Samman M, Marrodan M, María Pasquini J, Correale J (2022) 2-Chlorodeoxyadenosine (Cladribine) preferentially inhibits the biological activity of microglial cells. Int Immunopharmacol 105:108571 35093689 10.1016/j.intimp.2022.108571

[39] Jørgensen L, Hyrlov KH, Elkjaer ML, Weber AB, Pedersen AE, Svenningsen ÅF, Illes Z (2020) Cladribine modifies functional properties of microglia. Clin Exp Immunol 201:328–340 32492189 10.1111/cei.13473 PMC7419928

[40] Schmierer K, Vermersch P, Wiendl H, Montalban X, Achiron A, Derfuss T, Chan A, Prat A, Leocani L, Sellebjerg F, Hodgkinson S, Petit C, Nolting A, Gardner L, De Stefano N (2025) Durable effects of cladribine tablets on CSF OCB and NfL over 4 years in relapsing multiple sclerosis: Results from the MAGNIFY-MS Extension Study. Presented at ACTRIMS 2025 Forum. 27 February 1-March 2025

[41] Vermersch P, Wiendl H, Barkhof F, Montalban X, Achiron A, Derfuss T, Chan A, Hodgkinson S, Prat A, Leocani L, Schmierer K, Sellebjerg F, Gardner L, Petit C, Chudecka A, De Stefano N (2024) Improved cognitive processing speed in patients treated with cladribine tablets for multiple sclerosis: MAGNIFY-MS 2-year findings. Presented at ACTRIMS 2024 Forum. 29 February -2 March 2024

[42] De Stefano N, Vermersch P, Wiendl H, Barkhof F, Montalban X, Achiron A, Derfuss T, Chan A, Prat A, Leocani L, Schmierer K, Sellebjerg F, Lehn A, Smyk A, Nolting A, Koelbach R, Hodgkinson S (2024) Long-term effectiveness of cladribine tablets over 4 years in relapsing multiple sclerosis: Results from the MAGNIFY-MS Extension study. Multiple Sclerosis and Related Disorders 92:106123

[43] Brochet B, Solari A, Lechner-Scott J, Piehl F, Langdon D, Hupperts R, Selmaj K, Patti F, Brieva L, Maida EM, Alexandri N, Smyk A, Nolting A, Keller B, Montalban X, Kubala Havrdova E (2023) Improvements in quality of life over 2 years with cladribine tablets in people with relapsing multiple sclerosis: the CLARIFY-MS study. Mult Scler 29:1808–1818 37978852 10.1177/13524585231205962 PMC10687821

[44] Langdon D, Brochet B, Kubala Havrdova E, Patti F, Montalban X, Lechner-Scott J, Smyk A, Danten M, Selmaj K, Solari A (2025) Improvements in health-related quality of life and preserved cognitive function in patients with relapsing multiple sclerosis: 4‑year results from the CLARIFY-MS extension study. Presented at ACTRIMS Forum 2025. Feb 27-Mar 1, 2025

[45] Raji A, G W (2023) Cladribine tablets in highly active MS monitored by global and regional brain volumetry. Presented at ECTRIMS. October, 11–13, 2023

[46] Cortese R, Battaglini M, Sormani MP, Luchetti L, Gentile G, Inderyas M, Alexandri N, De Stefano N (2023) Reduction in grey matter atrophy in patients with relapsing multiple sclerosis following treatment with cladribine tablets. Eur J Neurol 30:179–186 36168741 10.1111/ene.15579 PMC10091690

[47] De Stefano N, Wiendl H, Vermersch P, Derfuss T, Montalban X, Achiron A, Hodgkinson S, Chan A, Prat A, Leocani L, Schmierer K, Sellebjerg F, Petit C, Helman A, Gardner L, Barkhof F (2025) Relationship between SDMT and brain volume following short course cladribine tablets: Results from MAGNIFY-MS. Presented at European Academy of Neurology (EAN 2025) 21–24 June 2025

[48] Marrodan M, Yañez P, Calandri IL, Piedrabuena MA, Zárate MA, Ysrraelit MC, Fiol M, Correale J (2025) Impact of oral Cladribine on paramagnetic rim lesions of Multiple Sclerosis patients. Mult Scler Relat Disord 96:106339 40020453 10.1016/j.msard.2025.106339

[49] Dal-Bianco A, Oh J, Sati P, Absinta M (2024) Chronic active lesions in multiple sclerosis: classification, terminology, and clinical significance. Ther Adv Neurol Disord 17:17562864241306684 39711984 10.1177/17562864241306684 PMC11660293

[50] Haider L, Simeonidou C, Steinberger G, Hametner S, Grigoriadis N, Deretzi G, Kovacs GG, Kutzelnigg A, Lassmann H, Frischer JM (2014) Multiple sclerosis deep grey matter: the relation between demyelination, neurodegeneration, inflammation and iron. J Neurol Neurosurg Psychiatry 85:1386–1395 24899728 10.1136/jnnp-2014-307712 PMC4251183

[51] De Stefano N, Wiendl H, Barkhof F, Achiron A, Derfuss T, Chan A, Hodgkinson S, Prat A, Leocani L, Schmierer K, Sellebjerg F, Vermersch P, Petit C, Gardner L, Montalban X (2024) Low rate of progression independent of relapse activity (PIRA) in patients with relapsing multiple sclerosis treated with cladribine tablets. Presented at ECTRIMS. September 18–20, 2024

[52] Giovannoni G, Comi G, Rammohan K, Rieckmann P, Dangond F, Keller B, Jack D, Vermersch P (2021) Long-term disease stability assessed by the Expanded Disability Status Scale in patients treated with cladribine tablets 3.5 mg/kg for relapsing multiple sclerosis: an exploratory post hoc analysis of the CLARITY and CLARITY Extension studies. Adv Ther 38:4975–4985 34370275 10.1007/s12325-021-01865-w PMC8408069

[53] Magalashvili D, Mandel M, Dreyer-Alster S, Didikin M, Harari G, Flechter S, Achiron A (2022) Cladribine treatment for highly active multiple sclerosis: real-world clinical outcomes for years 3 and 4. J Neuroimmunol 372:577966 36162338 10.1016/j.jneuroim.2022.577966

[54] Leist T, Cook S, Comi G, Montalban X, Giovannoni G, Nolting A, Damian D, Syed S, Galazka A (2020) Long-term safety data from the cladribine tablets clinical development program in multiple sclerosis. Mult Scler Relat Disord 46:102572 33296971 10.1016/j.msard.2020.102572

[55] Giovannoni G, Leist T, Aydemir A, Cantogno EVD (2022) Long-Term efficacy for patients receiving cladribine tablets in CLARITY/CLARITY extension: primary results from 9–15 years of follow-up in the CLASSIC-MS study. Mult Scler Relat Disord 59:103633

[56] Reitano P, Chisari CG, Patti F (2025) New strategies to manage the safety of cladribine in patients with multiple sclerosis. Expert Opin Drug Saf 24:389–394 39750104 10.1080/14740338.2024.2448826

[57] Hellwig K, Tilson HH, Thiel S, Ball K, Seebeck J, Danten M, Dubois N, Sabidó M (2025) Pregnancy and infant outcomes in multiple sclerosis: findings from the Global MAPLE-MS Pharmacovigilance Program. Neurol Neuroimmunol Neuroinflamm 12:e200438 40587835 10.1212/NXI.0000000000200438 PMC12221160

[58] Rolfes L, Pfeuffer S, Skuljec J, He X, Su C, Oezalp SH, Pawlitzki M, Ruck T, Korsen M, Kleinschnitz K, Aslan D, Hagenacker T, Kleinschnitz C, Meuth SG, Pul R (2023) Immune response to seasonal influenza vaccination in multiple sclerosis patients receiving cladribine. Cells. 10.3390/cells12091243 37174643 10.3390/cells12091243 PMC10177067

[59] Grothe C, Steffen F, Bittner S (2021) Humoral immune response and lymphocyte levels after complete vaccination against COVID-19 in a cohort of multiple sclerosis patients treated with cladribine tablets. J Cent Nerv Syst Dis 13:11795735211060118 34880703 10.1177/11795735211060118 PMC8647228

[60] Brill L, Rechtman A, Zveik O, Haham N, Levin N, Shifrin A, Rozenberg A, Vaknin-Dembinsky A (2022) Effect of cladribine on COVID-19 serology responses following two doses of the BNT162b2 mRNA vaccine in patients with multiple sclerosis. Mult Scler Relat Disord 57:103343 35158452 10.1016/j.msard.2021.103343 PMC8539216

[61] EMA Mavenclad: EPAR - Product Information (May 2025). Available at: https://www.ema.europa.eu/en/documents/product-information/mavenclad-epar-product-information_en.pdf. Accessed: 15 Jul 2025

[62] Hellwig K, Tokic M, Thiel S, Esters N, Spicher C, Timmesfeld N, Ciplea AI, Gold R, Langer-Gould A (2022) Multiple Sclerosis Disease Activity and Disability Following Discontinuation of Natalizumab for Pregnancy. JAMA Netw Open 5:e2144750 35072719 10.1001/jamanetworkopen.2021.44750 PMC8787598

[63] Hellwig K, Tokic M, Thiel S, Hemat S, Timmesfeld N, Ciplea AI, Gold R, Langer-Gould AM (2023) Multiple sclerosis disease activity and disability following cessation of fingolimod for pregnancy. Neurol Neuroimmunol Neuroinflamm. 10.1212/NXI.0000000000200110 37217309 10.1212/NXI.0000000000200110 PMC10202774

[64] Dost-Kovalsky K, Thiel S, Ciplea AI, Gold R, Hellwig K (2023) Cladribine and pregnancy in women with multiple sclerosis: the first cohort study. Mult Scler 29:461–465 36278327 10.1177/13524585221131486

[65] Leist T, Yamout B, Harlow D, Javor A, Galazka A, Seebeck J (2024) Post-approval safety of cladribine tablets in the treatment of patients with multiple sclerosis: 2024 update. Presented at 9th MENACTRIMS Congress. November 1–2, 2024

[66] Freedman MS, Leist TP, Comi G, Cree BA, Coyle PK, Hartung HP, Vermersch P, Damian D, Dangond F (2017) The efficacy of cladribine tablets in CIS patients retrospectively assigned the diagnosis of MS using modern criteria: results from the ORACLE-MS study. Mult Scler 3:2055217317732802 10.1177/2055217317732802 PMC5637982 29051829

[67] Leist TP, Comi G, Cree BA, Coyle PK, Freedman MS, Hartung HP, Vermersch P, Casset-Semanaz F, Scaramozza M (2014) Effect of oral cladribine on time to conversion to clinically definite multiple sclerosis in patients with a first demyelinating event (ORACLE MS): a phase 3 randomised trial. Lancet Neurol 13:257–267 24502830 10.1016/S1474-4422(14)70005-5

[68] Meca-Lallana V, García Domínguez JM, López Ruiz R, Martín-Martínez J, Arés Luque A, Hernández Pérez MA, Prieto González JM, Landete Pascual L, Sastre-Garriga J (2022) Expert-agreed practical recommendations on the use of cladribine. Neurol Ther 11:1475–1488 36068429 10.1007/s40120-022-00394-0 PMC9447968

[69] Oh J, Ayer M, Alroughani R, Lemieux C, Morgan K, D’Eramo M, Vella T, Boshra A, de Souza S, di Verdun Cantogno E, Sabidó M (2022) High adherence and minimal delays of year 2 treatment in people with multiple sclerosis treated with cladribine tablets: results from multi-country patient support programmes. Multiple Sclerosis Related Disorders 71:104288

[70] Okuda DT, Livingston T, Moog TM, Smith AD, Lebson L, Piette E (2024) Real-World outcomes of people with relapsing multiple sclerosis switching from natalizumab or ocrelizumab to cladribine tablets. Presented at ACTRIMS Forum 2024. 29 February 2-March 2024

[71] O’Neill D, Sharma M, Dong G, Vandenheuvel M (2023) Hodgkinson S (2023) Switching from ocrelizumab to cladribine: Real-world evidence. Multiple Sclerosis Related Disorders 69:104459 36565573 10.1016/j.msard.2022.104459

[72] Ellenberger D, Frahm N, Flachenecker P, Hellwig K, Kleinschnitz C, Paul F, Warnke C, Übler S, Stahmann A (2022) Treatment patterns prior to and post cladribine in patients with multiple sclerosis. Eur J Neurol 29:629–630

[73] Sacco R, Disanto G, Pravatà E, Mallucci G, Maceski AM, Kuhle J, Gobbi C, Zecca C (2024) De-escalation from anti-CD20 to cladribine tablets in multiple sclerosis: a pilot study. Mult Scler Relat Disord 92:106145 39510011 10.1016/j.msard.2024.106145

[74] Konen FF, Pfeuffer S, Jendretzky KF, Gehring K, Elias-Hamp B, Sühs K-W, Pawlitzki M, Meuth SG, Kleinschnitz C, Pul R, Skripuletz T (2024) Retrospective data analysis of a German cohort of patients with active relapsing multiple sclerosis switching from anti-CD20 therapies to cladribine tablets and vice versa. Presented at ECTRIMS 2024. 18-20 September 2024

[75] Wallin MT, Culpepper WJ, Campbell JD, Nelson LM, Langer-Gould A, Marrie RA, Cutter GR, Kaye WE, Wagner L, Tremlett H, Buka SL, Dilokthornsakul P, Topol B, Chen LH, LaRocca NG (2019) The prevalence of MS in the United States: a population-based estimate using health claims data. Neurology 92:e1029–e1040 30770430 10.1212/WNL.0000000000007035 PMC6442006

[76] Grebenciucova E, Berger JR (2017) Immunosenescence: the role of aging in the predisposition to neuro-infectious complications arising from the treatment of multiple sclerosis. Curr Neurol Neurosci Rep 17:61 28669032 10.1007/s11910-017-0771-9

[77] Coerver EME, Fung WH, de Beukelaar J, Bouvy WH, Canta LR, Gerlach OHH, Hoitsma E, Hoogervorst ELJ, de Jong BA, Kalkers NF, van Kempen ZLE, Lövenich H, van Munster CEP, van Oosten BW, Smolders J, Vennegoor A, Zeinstra E, Barrantes-Cepas M, Kooij G, Schoonheim MM, Lissenberg-Witte BI, Teunissen CE, Moraal B, Barkhof F, Uitdehaag BMJ, Mostert J, Killestein J, Strijbis EMM (2025) Discontinuation of first-line disease-modifying therapy in patients with stable multiple sclerosis: the DOT-MS randomized clinical trial. JAMA Neurol 82:123–131 39652340 10.1001/jamaneurol.2024.4164 PMC11811793

[78] Corboy JR, Fox RJ, Kister I, Cutter GR, Morgan CJ, Seale R, Engebretson E, Gustafson T, Miller AE (2023) Risk of new disease activity in patients with multiple sclerosis who continue or discontinue disease-modifying therapies (DISCOMS): a multicentre, randomised, single-blind, phase 4, non-inferiority trial. Lancet Neurol 22:568–577 37353277 10.1016/S1474-4422(23)00154-0

[79] Jouvenot G, Courbon G, Lefort M, Rollot F, Casey R, Le Page E, Michel L, Edan G, de Seze J, Kremer L, Bigaut K, Vukusic S, Mathey G, Ciron J, Ruet A, Maillart E, Labauge P, Zephir H, Papeix C, Defer G, Lebrun-Frenay C, Moreau T, Laplaud DA, Berger E, Stankoff B, Clavelou P, Thouvenot E, Heinzlef O, Pelletier J, Al-Khedr A, Casez O, Bourre B, Cabre P, Wahab A, Magy L, Camdessanché JP, Doghri I, Moulin S, Ben-Nasr H, Labeyrie C, Hankiewicz K, Neau JP, Pottier C, Nifle C, Collongues N, Kerbrat A (2024) High-efficacy therapy discontinuation vs continuation in patients 50 years and older with nonactive MS. JAMA Neurol 81:490–498 38526462 10.1001/jamaneurol.2024.0395 PMC10964164

[80] Coerver E, Schoof L, Hogenboom L, Wessels M, van Ruyven P, van Samkar A, Mostert J, van Kempen Z, van Oosten BW, Wokke BH, Tallantyre E, Myhr KM, Torkildsen O, Killestein J, Smets I, Strijbis E (2024) The recurrence of disease activity after ocrelizumab discontinuation in multiple sclerosis. Mult Scler Relat Disord 91:105900 39369631 10.1016/j.msard.2024.105900

[81] Rammohan K, Giovannoni G, Comi G, Cook S, Rieckmann P, Soelberg Sørensen P, Vermersch P, Hamlett A, Kurukulasuriya N (2012) Cladribine tablets for relapsing-remitting multiple sclerosis: efficacy across patient subgroups from the phase III CLARITY study. Mult Scler Relat Disord 1:49–54 25876451 10.1016/j.msard.2011.08.006

[82] Roos I, Sharmin S, Müller J, Horakova D, Havrdova E, Ozakbas S, Prat A, Girard M, Duquette P, Libertínová J, Mares M, Grammond P, Prévost J, Rous Z, Lechner Scott J, Buzzard K, Skibina O, Hradilek P, van der Walt A, Butzkueven H, Boz C, Patti F, Barnett M, Ayuso GI, Eichau S, Blanco Y, Meca-Lallana JE, Foschi M, Surcinelli A, Recmanova E, Gerlach O, Kuhle J, Terzi M, Pavelek Z, John NA, McCombe P, Yamout BI, Khoury S, Turkoglu R, D'amico E, Ampapa R, Stourac P, Kermode A, Pedrini M, Carroll W, MacDonell R, Cartechini E, Slee M, Guimaraes J, Peterka M, Castillo-Triviño T, Van Pesch V, Laureys G, Houskova J, Sanchez JL, Ramo Tello C, Alroughani R, Soysal A, Hodgkinson S, Kalincik T (2025) Cladribine as an exit strategy in people with MS over the age of 50. Presented at ECTRIMS 2025. September 24-26, 2025

[83] Achiron A, Mandel M, Dreyer-Alster S, Harari G, Dolev M, Menascu S, Magalashvili D, Flechter S, Givon U, Guber D, Sonis P, Zilkha-Falb R, Gurevich M (2021) Humoral immune response in multiple sclerosis patients following PfizerBNT162b2 COVID19 vaccination: up to 6 months cross-sectional study. J Neuroimmunol 361:577746 34655991 10.1016/j.jneuroim.2021.577746 PMC8500842

[84] Baker D, Herrod SS, Alvarez-Gonzalez C, Zalewski L, Albor C, Schmierer K (2017) Both cladribine and alemtuzumab may effect MS via B-cell depletion. Neurol Neuroimmunol Neuroinflamm 4:e360 28626781 10.1212/NXI.0000000000000360 PMC5459792

[85] Baker D, MacDougall A, Kang AS, Schmierer K, Giovannoni G, Dobson R (2022) CD19 B cell repopulation after ocrelizumab, alemtuzumab and cladribine: implications for SARS-CoV-2 vaccinations in multiple sclerosis. Mult Scler Relat Disord 57:103448 34902760 10.1016/j.msard.2021.103448 PMC8642825

[86] Pröbstel AK, Hauser SL (2018) Multiple sclerosis: B cells take center stage. J Neuroophthalmol 38:251–258 29561328 10.1097/WNO.0000000000000642 PMC6505460

[87] Rolfes L, Pfeuffer S, Huntemann N, Schmidt M, Su C, Skuljec J, Aslan D, Hackert J, Kleinschnitz K, Hagenacker T, Pawlitzki M, Ruck T, Kleinschnitz C, Meuth SG, Pul R (2022) Immunological consequences of cladribine treatment in multiple sclerosis: a real-world study. Mult Scler Relat Disord 64:103931 35690010 10.1016/j.msard.2022.103931

[88] Wiendl H, Schmierer K, Hodgkinson S, Derfuss T, Chan A, Sellebjerg F, Achiron A, Montalban X, Prat A, De Stefano N, Barkhof F, Leocani L, Vermersch P, Chudecka A, Roy S, Boschert U (2021) Characterization of peripheral immune cell dynamics and repopulation patterns in the first 12 months of cladribine tablets treatment: MAGNIFY-MS study (2235). Neurology 96:2235

[89] Waldrop G, Sisodia N, Poole S, Pleasure S, Wilson MR, Guo CY, Gelfand JM, Zamvil SS, Bove R (2025) Neutropenia Associated With B Cell-Depleting Therapies in Multiple Sclerosis and Neuromyelitis Optica Spectrum Disorder. Neurol Neuroimmunol Neuroinflamm 12:e200430 40537081 10.1212/NXI.0000000000200430 PMC12185220

[90] Baker D, Kang AS, Giovannoni G, Schmierer K (2024) Neutropenia following immune-depletion, notably CD20 targeting, therapies in multiple sclerosis. Mult Scler Relat Disord 82:105400 38181696 10.1016/j.msard.2023.105400

[91] Dukkipati R (2024) Durable efficacy of cladribine tablets in a US real-world population. Presented at ACTRIMS Forum 2024. 29 February - 2 March 2024

[92] Roos I, Malpas C, Leray E, Casey R, Horakova D, Havrdova EK, Debouverie M, Patti F, De Seze J, Izquierdo G, Eichau S, Edan G, Prat A, Girard M, Ozakbas S, Grammond P, Zephir H, Ciron J, Maillart E, Moreau T, Amato MP, Labauge P, Alroughani R, Buzzard K, Skibina O, Terzi M, Laplaud DA, Berger E, Grand’Maison F, Lebrun-Frenay C, Cartechini E, Boz C, Lechner-Scott J, Clavelou P, Stankoff B, Prevost J, Kappos L, Pelletier J, Shaygannejad V, Yamout BI, Khoury SJ, Gerlach O, Spitaleri DLA, Van Pesch V, Gout O, Turkoglu R, Heinzlef O, Thouvenot E, McCombe PA, Soysal A, Bourre B, Slee M, Castillo-Trivino T, Bakchine S, Ampapa R, Butler EG, Wahab A, Macdonell RA, Aguera-Morales E, Cabre P, Ben NH, Van der Walt A, Laureys G, Van Hijfte L, Ramo-Tello CM, Maubeuge N, Hodgkinson S, Sánchez-Menoyo JL, Barnett MH, Labeyrie C, Vucic S, Sidhom Y, Gouider R, Csepany T, Sotoca J, de Gans K, Al-Asmi A, Fragoso YD, Vukusic S, Butzkueven H, Kalincik T (2022) Disease reactivation after cessation of disease-modifying therapy in patients with relapsing-remitting multiple sclerosis. Neurology 99:e1926–e1944 35977837 10.1212/WNL.0000000000201029 PMC9620810

[93] Frau J, Sormani MP, Signori A, Realmuto S, Baroncini D, Annovazzi P, Signoriello E, Maniscalco GT, La Gioia S, Cordioli C, Frigeni B, Rasia S, Fenu G, Grasso R, Sartori A, Lanzillo R, Stromillo ML, Rossi S, Forci B, Cocco E (2018) Clinical activity after fingolimod cessation: disease reactivation or rebound? Eur J Neurol 25:1270–1275 29851435 10.1111/ene.13694

[94] Maunula A, Atula S, Laakso SM, Tienari PJ (2024) Frequency and risk factors of rebound after fingolimod discontinuation - a retrospective study. Mult Scler Relat Disord 81:105134 37980790 10.1016/j.msard.2023.105134

[95] Tunç A, Yetkin MF, Seferoğlu M, İnanç Y, Sıvacı A, Türkoğlu ŞA, Baydar C, Güzel V, Bülbül NG, Sezer V, Altun Y (2024) Recurring disease activity in relapsing remitting multiple sclerosis: the multicenter RDA-RMS study. Mult Scler Relat Disord 88:105757 38972107 10.1016/j.msard.2024.105757

[96] Zhu C, Zhou Z, Roos I, Merlo D, Kalincik T, Ozakbas S, Skibina O, Kuhle J, Hodgkinson S, Boz C, Alroughani R, Lechner-Scott J, Barnett M, Izquierdo G, Prat A, Horakova D, Kubala Havrdova E, Macdonell R, Patti F, Khoury SJ, Slee M, Karabudak R, Onofrj M, Van Pesch V, Prevost J, Monif M, Jokubaitis V, van der Walt A, Butzkueven H (2022) Comparing switch to ocrelizumab, cladribine or natalizumab after fingolimod treatment cessation in multiple sclerosis. J Neurol Neurosurg Psychiatry 93:1330–1337 36261289 10.1136/jnnp-2022-330104

[97] Nygaard GO, Torgauten H, Skattebøl L, Høgestøl EA, Sowa P, Myhr KM, Torkildsen Ø, Celius EG (2022) Risk of fingolimod rebound after switching to cladribine or rituximab in multiple sclerosis. Mult Scler Relat Disord 62:103812 35462167 10.1016/j.msard.2022.103812

[98] Ciron J, Bourre B, Castelnovo G, Guennoc AM, De Sèze J, Ben-Amor AF, Savarin C, Vermersch P (2024) Holistic, Long-Term Management of People with Relapsing Multiple Sclerosis with Cladribine Tablets: Expert Opinion from France. Neurol Ther 13:503–518 38488979 10.1007/s40120-024-00589-7 PMC11136930

[99] Sönmez MT, Yetkin MF, Mehdiyev DA, Köseoğlu M, Mungan S, Kale N, Terzi M, Tunç A, Koç ER, Şen S, Ozben S, Yoldaş TK, Çilingir V, Kotan D, Aksoy D, Aydın FK, Koçer B, Demir M, Çam M, Öztürk P, Fırat YE, Ömerhoca S, Ercan MB, Skuljec J, Pul R (2025) Safety and efficacy of cladribine in patients discontinuing fingolimod due to elevated transaminase levels: the FinClad study. Mult Scler Relat Disord 101:106578 40570400 10.1016/j.msard.2025.106578

[100] Gasperini C, Centonze D, Conte A, Gallo P, Lugaresi A, Patti F, Trojano M, Amato MP, Filippi M (2025) Personalized therapy in multiple sclerosis: an Italian Delphi consensus. J Neurol 272:428 40423800 10.1007/s00415-025-13173-2 PMC12116865

[101] Polistena B, Provenzano AM, Rizzi C, Colombo E, Bergamaschi R (2025) Cost-consequences of cladribine tablets for the treatment of highly active relapsing-remitting multiple sclerosis in Italy. Neurol Ther 14:1507–1520 40461934 10.1007/s40120-025-00761-7 PMC12255608

[102] Tafazzoli A, Chavan A, Harty G, Moller J, Wong SL (2020) Efficiency model of cladribine tablets versus infusion-based disease-modifying drugs for patients with relapsing-remitting multiple sclerosis. Adv Ther 37:3791–3806 32647909 10.1007/s12325-020-01426-7

[103] Mankinen P, Lundström T, Soini E, Sumelahti ML, Ruutiainen J, Niskala U, Järvinen E (2020) Cost assessment modelling of treatments for highly active relapsing multiple sclerosis. Adv Ther 37:800–818 31873868 10.1007/s12325-019-01186-z PMC6999169

[104] Poveda JL, Trillo JL, Rubio-Terrés C, Rubio-Rodríguez D, Polanco A, Torres C (2020) Cost-effectiveness of cladribine tablets and fingolimod in the treatment of relapsing multiple sclerosis with high disease activity in Spain. Expert Rev Pharmacoecon Outcomes Res 20:295–303 31220959 10.1080/14737167.2019.1635014

[105] Versteegh MM, Huygens SA (2025) Exit strategies in patients with stable MS: cost-effectiveness of extended interval dosing of ocrelizumab and natalizumab versus de-escalating to cladribine. Mult Scler Relat Disord 102:106625 40714725 10.1016/j.msard.2025.106625

[106] Montalban X, Lebrun-Frénay C, Oh J, Arrambide G, Moccia M, Pia Amato M, Amezcua L, Banwell B, Bar-Or A, Barkhof F, Butzkueven H, Ciccarelli O, Chataway J, Cohen JA, Comi G, Correale J, Deisenhammer F, Filippi M, Fiol J, Freedman MS, Fujihara K, Granziera C, Green AJ, Hartung HP, Hellwig K, Kappos L, Kimbrough D, Killestein J, Lublin F, Marignier R, Ann Marrie R, Miller A, Otero-Romero S, Ontaneda D, Ramanathan S, Reich D, Rocca MA, Rovira À, Saidha S, Salter A, Sastre-Garriga J, Saylor D, Solomon AJ, Sormani MP, Stankoff B, Tintore M, Tremlett H, Van der Walt A, Viswanathan S, Wiendl H, Wildemann B, Yamout B, Zaratin P, Calabresi PA, Coetzee T, Thompson AJ (2025) Diagnosis of multiple sclerosis: 2024 revisions of the McDonald criteria. Lancet Neurol 24:850–865 40975101 10.1016/S1474-4422(25)00270-4

[107] Wiendl H, Derfuss T, Sponton L, Jin H, Fluck M, Guala D, Gardner L, De Stefano N, Leocani L (2025) Impact of cladribine tablets on GFAP levels and intrathecal biomarkers in RMS: Results from the MAGNIFY-MS study. Presented at ECTRIMS 2025. September 24-26, 2025

